# Several fusion genes identified by whole transcriptome sequencing in a spindle cell sarcoma with rearrangements of chromosome arm 12q and *MDM2* amplification

**DOI:** 10.3892/ijo.2014.2605

**Published:** 2014-08-18

**Authors:** IOANNIS PANAGOPOULOS, BODIL BJERKEHAGEN, LUDMILA GORUNOVA, JEANE-MARIE BERNER, KJETIL BOYE, SVERE HEIM

**Affiliations:** 1 Section for Cancer Cytogenetics, Institute for Cancer Genetics and Informatics, The Norwegian Radium Hospital, Oslo University Hospital, Oslo, Norway; 2 Centre for Cancer Biomedicine, Faculty of Medicine, University of Oslo, Oslo, Norway; 3 Department of Pathology, The Norwegian Radium Hospital, Oslo University Hospital, Oslo, Norway; 4 Department of Oncology, The Norwegian Radium Hospital, Oslo University Hospital, Oslo, Norway; 5 Department of Tumor Biology, Institute for Cancer Research, The Norwegian Radium Hospital, Oslo University Hospital, Oslo, Norway; 6 Faculty of Medicine, University of Oslo, Oslo, Norway

**Keywords:** fusion genes, whole transcriptome sequencing, spindle cell sarcoma, MDM2 amplification, rearrangements of chromosome Arm 12q, *PTGES3-PTPRB*, *HMGA2-DYRK2*, *TMBIM4-MSRB3*, *USP15-CNTN1*

## Abstract

Spindle cell tumors are clinically heterogeneous but morphologically similar neoplasms that can occur anywhere, mostly in adult patients. They are treated primarily with surgery to which is often added adjuvant or neoadjuvant radiation. Sub-classification of spindle cell sarcomas requires integration of histology, clinicopathological parameters, immunohistochemistry, cytogenetics (including fluorescence *in situ* hybridization) and/or molecular genetics. Some of the tumor subtypes are characterized by the presence of distinct chromosomal translocations and fusion genes. When no signs of differentiation are seen, the diagnosis by exclusion becomes undifferentiated spindle cell sarcoma. Cytogenetic, RNA sequencing and RT-PCR analyses were performed on a case of spindle cell sarcoma. The karyotype of the primary tumor was 46,X,del(X)(p?11p?22), der(12)(12pter→12q?22::12q?15→ q?22::16p11→16pter),-16,+r(12). *MDM2* was found amplified in both the primary tumor and a metastasis. RNA-Seq of the primary tumor identified four fusion genes, *PTGES3-PTPRB*, *HMGA2-DYRK2*, *TMBIM4-MSRB3* and *USP15-CNTN1*, in which all the partner genes map to the q arm of chromosome 12. In material from the metastasis, RT-PCR detected the *PTGES3-PTPRB*, *HMGA2-DYRK2* and *TMBIM4-MSRB3* whereas no *USP15-CNTN1* fusion transcript was found. Because *MDM2* amplification and the fusion transcripts *PTGES3-PTPRB*, *HMGA2-DYRK2* and *TMBIM4-MSRB3* were found both in the primary tumor and in the metastasis, they are components of the same clone and may be involved both in initiation and progression of the tumor. Which of them is pathogenetically primary remains unknown.

## Introduction

Spindle cell tumors are clinically heterogeneous neoplasms with similar morphology. The term is descriptive and based on the tumor cells’ long and slender microscopic appearance. The tumor may be a true sarcoma (connective tissue cancer) or only sarcomatoid which means a tumor that looks like a sarcoma under the microscope (http://www.cancer.org/index). Spindle cell sarcomas can occur anywhere. They are more common in adults and are treated primarily with surgery to which is often added adjuvant or neoadjuvant radiation (1). Sub-classification of spindle cell sarcomas requires integration of histology, clinicopathological parameters, immunohistochemistry, cytogenetics (including fluorescence *in situ* hybridization) and/or molecular genetics. When no identifiable differentiation can be made the diagnosis is undifferentiated spindle cell sarcoma by exclusion (2). Other tumors are subdivided depending on the differentiation they show. The latter group includes leiomyosarcoma (smooth muscle differentiation), malignant peripheral nerve sheath tumor, fibrosarcoma (fibroblastic differentiation) and myofibroblastic sarcoma (myofibroblastic differentiation) among others. In the subgroup with uncertain differentiation, synovial sarcoma is the most common (1). Cytogenetic and molecular genetic analyses of spindle cell sarcomas have led to the recognition of several distinct chromosomal translocations and fusion genes that characterize specific tumor types. For example, synovial sarcomas carry the translocation t(X;18)(p11;q11) in more than 95% of the cases. It results in rearrangement of the *SS18* gene in 18q11 with one of the *SSX1*, *SSX2* or *SSX4* genes in Xp11 creating an *SS18-SSX1*, *SS18-SSX2* or *SS18-SSX4* chimeric gene (3). A subset of inflammatory myofibroblastic tumor, a neoplasm composed of myofibroblastic spindle cells and infiltrating inflammatory cells, harbors clonal chromosomal rearrangements involving chromosome band 2p23. These rearrangements target the *ALK* gene, serving as the 3′-partner in fusions with various translocation partners and activating ALK tyrosine kinase function (3). Dermatofibrosarcoma protuberans, another subtype of spindle cell sarcoma, is characterized cytogenetically either by supernumerary ring chromosomes (60% of all examined tumors) or by the translocation t(17;22)(q22;q13) (http://cgap.nci.nih.gov/Chromosomes/Mitelman). Either event results in the formation of a *COL1A1-PDGFB* fusion gene in which *PDGFB* exon 1 is deleted and replaced by a variable segment of the *COL1A1* gene (4). Solitary fibrous tumors are distinguished by their hemangiopericytoma-like branching vascular pattern, positivity for CD34 by immunohistochemistry and *NAB2-STAT6* fusion (2,5,6). Further sub-classification of spindle cell sarcomas may in the future be of value but is currently difficult. Eyden *et al* (7) presented five ‘diagnostically problematic spindle cell sarcomas’ which lacked defined line of differentiation by light microscopy and showed different clinical courses. In a correlation study between clinicopathological features and karyotypes in spindle cell sarcomas, Fletcher *et al* (8) reported three unclassified spindle cell sarcomas, two of which showed a normal karyotype whereas the third had a complex karyotype with multiple aberrations. Alaggio *et al* (9) described two spindle cell tumors with *EWSR1-WT1* fusion gene and favorable prognosis. According to the authors, these tumors could represent ‘an unrecognized subgroup of tumors with spindle cell morphology, bearing the same translocation as desmoplastic small round cell tumor, but characterized by a more favorable clinical course’ (9). Lestou *et al* (10) reported a case of spindle cell sarcoma with a complex karyotype and ring chromosomes. Further analysis with multicolor fluorescence *in situ* hybridization (FISH) probes showed amplification of chromosome 18 and co-amplification of 12p11 and 12q13~q22 in the ring chromosomes. Recently, Nord *et al* (11) reported a spindle cell sarcoma of the heart with ring chromosome, amplification of the *MDM2* gene and homozygous deletion of *CDKN2A* (11).

In the present study, we describe a spindle cell sarcoma with ring chromosome composed of chromosome 12 material, several fusion genes mapping to 12q, and amplification of *MDM2*.

## Materials and methods

### Ethics statement

The study was approved by the Regional Committee for Medical Research Ethics (Regional komité for medisinsk forskningsetikk Sør-Øst, Norge, http://helseforskning.etikkom.no).

### Case report

**Figure 1:**
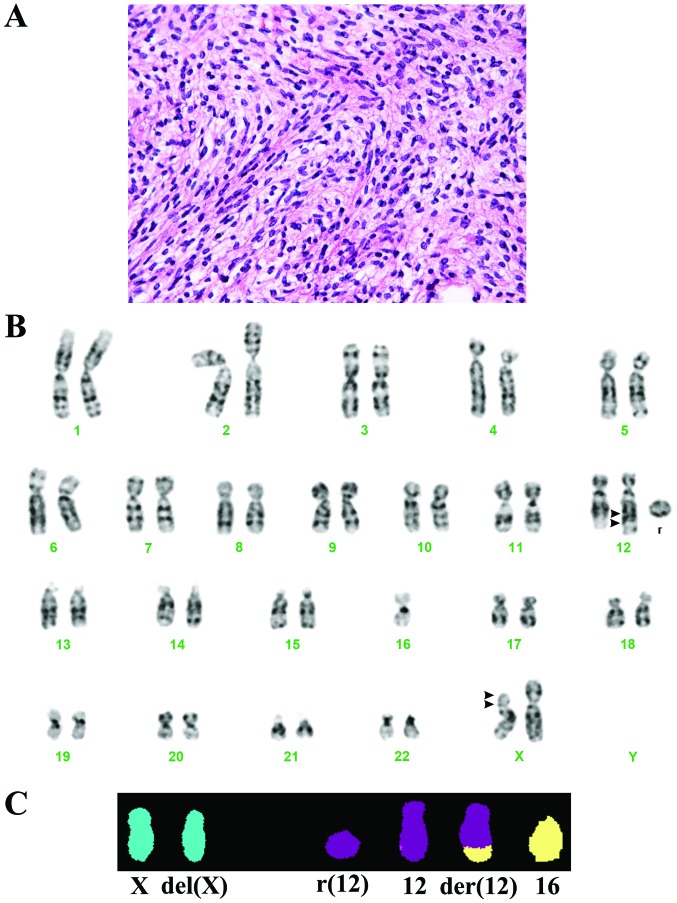
Microscopic examination, karyotype and M-FISH analyses of the primary tumor. (A) H&E-stained section of the tumor. (B) Karyotype at diagnosis showing the chromosome aberrations del(X)(p?11p?22), der(12)(12pter→12q?22::12q?15→q?22::16p11→16pter),−16 and r(12). (C) M-FISH showing that the del(X) chromosome does not contain material from other chromosomes, the ring chromosome is composed of chromosome 12 material and der(12) contains material from chromosome 16. Arrowheads indicate chromosome breakpoints.

The patient was a 69-year-old woman who presented with right-sided chest pain and reduced general condition. Clinical and radiological work-up revealed a large tumor in the lower lobe of the right lung. No metastases were detected. After a needle biopsy, bilobectomy was performed and the tumor was removed with adequate margins. Macroscopic examination showed a well demarcated 11 cm large tumor. Microscopic examination disclosed a cellular tumor with spindle cells and some round cells with slight atypia surrounded by loose intercellular substance and some collagen (Fig. 1A). No atypical lipoblasts or pleomorphic cells were seen. There was no necrosis. The mitotic count was 8/10HPF and the French malignancy grade 2 (2). Immunohistochemical analysis showed negative staining for AE1/AE3, CK5/6, EMA, CD31, CD34, CD45, CD117, CD99, actin, smooth-muscle actin, desmin, h-caldesmon, calretinin, HM B-45 and protein S-100. RT-PCR was negative for gene fusions consistent with synovial sarcoma. The morphological picture and immunohistochemical findings were consistent with a spindle cell sarcoma not otherwise specified. No adjuvant therapy was administered. Two-and-a-half years after primary surgery a systemic relapse was diagnosed, with soft tissue metastases in the retroperitoneum and the large adductor muscle of the left thigh, a solitary lung metastasis in the right upper lobe, a tumor in the pancreatic head and bone metastases in the 7th lumbar vertebra and the left pubic bone. A core needle biopsy of the tumor in the left thigh confirmed the diagnosis of metastatic spindle cell sarcoma. No radiotherapy or systemic treatment was given, and the patient died 15 months later of metastatic disease.

### G-banding, karyotyping and multicolor fluorescence in situ hybridization (mFISH)

A sample from the surgically removed primary tumor was mechanically and enzymatically disaggregated and short-term cultured as described elsewhere (12). The culture was harvested and the chromosomes G-banded using Wright stain. The subsequent cytogenetic analysis and karyotype description followed the recommendations of the ISCN (13). For multicolor FISH the 24XCyte Human Multicolor FISH Probe kit was used (MetaSystems-international, http://www.metasystems-international.com/) according to the manufacturer’s protocol. Fluorescent signals were captured and analyzed using the CytoVision system (Leica Biosystems, Newcastle, UK).

### High-throughput paired-end RNA-sequencing

Tumor tissue adjacent to that used for cytogenetic analysis and histological examination had been frozen and stored at −80°C. Total RNA was extracted from both primary tumor and the metastasis in the left thigh using TRIzol reagent according to the manufacturer’s instructions (Life Technologies, Oslo, Norway) and its quality was checked by Experion Automated Electrophoresis System (Bio-Rad Laboratories, Oslo, Norway). Three micrograms of total RNA from the primary tumor were sent for high-throughput paired-end RNA-sequencing at the Genomics Core Facility, The Norwegian Radium Hospital (http://genomics.no/oslo/). The RNA was sequenced using an Illumina HiSeq 2500 instrument and the Illumina software pipeline was used to process image data into raw sequencing data. Only sequence reads marked as ‘passed filtering’ were used in the downstream data analysis. A total of 120 million reads were obtained. The software FusionMap (release date 2012-04-16) and the associated pre-built Human B37 and RefGene from the FusionMap website (http://www.omicsoft.com/fusionmap/) were used for the discovery of fusion transcripts (14).

### Polymerase chain reaction

The primers used for PCR amplification and sequencing are listed in Table I. For Reverse Transcriptase-Polymerase Chain Reaction (RT-PCR), 1 μg of total RNA from both primary tumor and the metastasis was reverse-transcribed in a 20-μl reaction volume using iScript Advanced cDNA Synthesis kit for RT-qPCR according to the manufacturer’s instructions (Bio-Rad). The cDNA was diluted to 50 μl and 2 μl were used as templates in subsequent PCR assays. The 25 μl PCR-volume contained 12.5 μl of Premix Taq (Takara Bio Europe/SAS, Saint-Germain-en-Laye, France), 2 μl of diluted cDNA and 0.4 μM of each of the forward and reverse primers. The PCRs were run on a C-1000 Thermal cycler (Bio-Rad). The PCR conditions were: an initial denaturation at 94°C for 30 sec followed by 35 cycles of 7 sec at 98°C and 2 min at 68°C, and a final extension for 5 min at 68°C. The following primer sets were used to detect the fusion transcripts: PTGES3-416F1/PTPRB-1610R1 for (*PTGES3-PTPRB*), HM GA2-982F1/DYRK2-733R1 for (*HMGA2-DYRK2*), TMBIM4-25F1/MSRB3-501R for (*TMBIM4-MSRB3*) and USP15-655F1/CNTN1-413R for (*USP15-CNTN1*).

For the detection of the genomic hybrid fragments, extra-long PCR (XL PCR) was carried out using the XL PCR kit (Applied Biosystems, Life Technologies). XL PCR was performed in 50 μl of 1:3 diluted 3.3X XL buffer II, 1.1 mM Mg(OAc)_2_, 0.2 mM of each dNTP, 1 unit of rTth DNA polymerase, XL, 0.4 μM of each of the forward and reverse primers (Table I) and 300 ng tumor DNA (or 2 μl of the first PCR in nested PCR). After an initial denaturation for 1 min at 94°C, 32 cycles of 15 sec at 94°C and 10 min at 68°C were run followed by a final extension for 10 min at 72°C. For the detection of genomic *PTGES3-PTPTRB* fusion DNA fragment, the primers PTGES3-Int4-F1New and PTTRB-1602R were used. For possible genomic *HMGA2-DYRK2* and *TMBIM4-MSRB3*, nested XL PCR was performed. For genomic *HMGA2-DYRK2* amplification, the primers HMG A2-Int4-F1/DYRK2-709R1 and HMG A2-Int4-F2/DYRK2-651R were used for first and nested PCR, respectively. For genomic *TMBIM4-MSRB3* amplification, the primers TMBIM4-55F1New/MSRB3-350R1New and TMBIM4-121F1New/MSRB3-261R1New were used for first and nested PCR, respectively.

Four microliters of the PCR products were stained with GelRed (Biotium, Hayward, CA, USA), analyzed by electrophoresis through 1.0% agarose gel and photographed. The amplified fragments were excised from the gel, purified using the Qiagen gel extraction kit (Qiagen), and direct sequencing (Sanger sequencing) was performed using the light run sequencing service of GATC Biotech (http://www.gatc-biotech.com/en/sanger-services/lightrun-sequencing.html). The BLAST software (http://blast.ncbi.nlm.nih.gov/Blast.cgi) was used for computer analysis of sequence data.

### MDM2 amplification

Genomic tumor DNA was extracted from both primary tumor and metastasis paraffin-embedded samples using QIAamp DNA FFPE Tissue kit as recommended by the manufacturer (Qiagen, Hilden, Germany). Real-time PCR was performed using the ABI PRISM^®^ 7900HT Sequence Detection System (Life Technologies, Carlsbad, CA). Primers and probes for *MDM2* (on 12q15), CDK4 (on 12q14.1) and *ALB* (on 4q13.3, reference gene) were as described by Hostein *et al* (15). PCR amplification was performed in duplicates in 25 μl reaction volume containing 12 ng of genomic DNA, 300 nM of each primer, 100 nM of the probe and Ix TaqMan Universal PCR Master Mix (Life Technologies). For the amplification of *ALB*, MgCl_2_ was added to a final concentration of 400 nM. The gene copy numbers were calculated from standard curves constructed by amplification of normal blood DNA of the target genes *MDM2* and *CDK4* and the reference gene *ALB.* The level of amplification was determined as copy number of target gene (*MDM2* or *CDK4*)/copy number of reference gene (*ALB*). A pool of six paraffin-embedded lipomas (confirmed by karyotyping) was used as normal control and the osteosarcoma cell line SJSAI (ATCC CRL-2098) as a positive control cell line.

## Results

**Figure 2:**
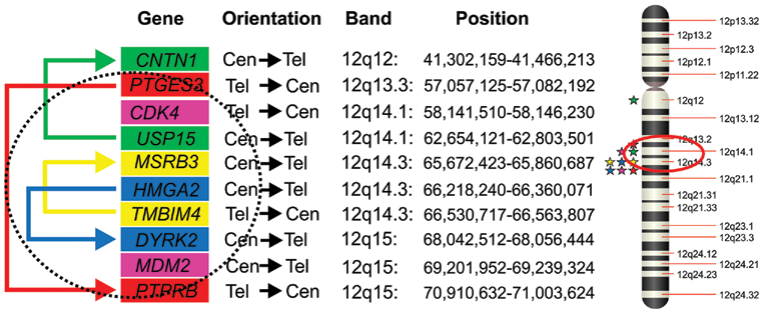
Diagram showing the genes involved in the fusions on the q arm of the chromosome 12, their orientations, locations and positions. The *CDK4* and *MDM2* genes are also included. The physical positions and the orientations of the genes are obtained from UCSC Genome Browser website (http://genome-euro.ucsc.edu/index.html) and they are based on the Human genome assembly of February 2009 (GRCh37/hg19). The colored arrows show the four fusion genes. The circle suggests a possible ring chromosome which leaves outside the *CNTN1* gene. This ring chromosome closes between *PTGES3* and *PTPRB* and would contain the *PTGES3-PTPRB*, *HMGA2-DYRK2* and *TMBIM4-MSRB3* as well as *CDK4* and the amplified *MDM2*. Stars indicate positions of the partner fusion genes on chromosome 12, the *CDK4* and *MDM2* genes.

The G-banding and M-FISH analyses yielded the karyotype 46,X,del(X)(p?11p?22), der(12)(12pter→12q?22::12q?15→q? 22::16p11→16pter),−16,+r(12)[23] (Fig. 1B and C). Using the FusionMap on the raw RNA sequencing data, approximately 500 potential fusion genes were found (data not shown). The *PTGES3-PTPRB*, *HMGA2-DYRK2*, *USP15-CNTN1* and *TMBIM4-MSRB3* fusion transcripts were ranked 1st with 60 seed counts, 3rd with 27 seed counts, 4th with 26 seed counts and 5th with 22 seed counts, respectively. We decided to investigate further these fusion genes in materials obtained from both the primary tumor and the metastasis because: i) all the partner genes in the above-mentioned fusion transcripts map to the q arm of chromosome 12 (Fig. 2) and ii) the karyotype of the primary tumor had a ring chromosome composed of chromosome 12 material and a translocation involving chromosomes 12 and 16. A potential read through sequence, *C10orf68-CCDC7*, ranked 2nd with 50 seed counts, was not further studied.

**Figure 3:**
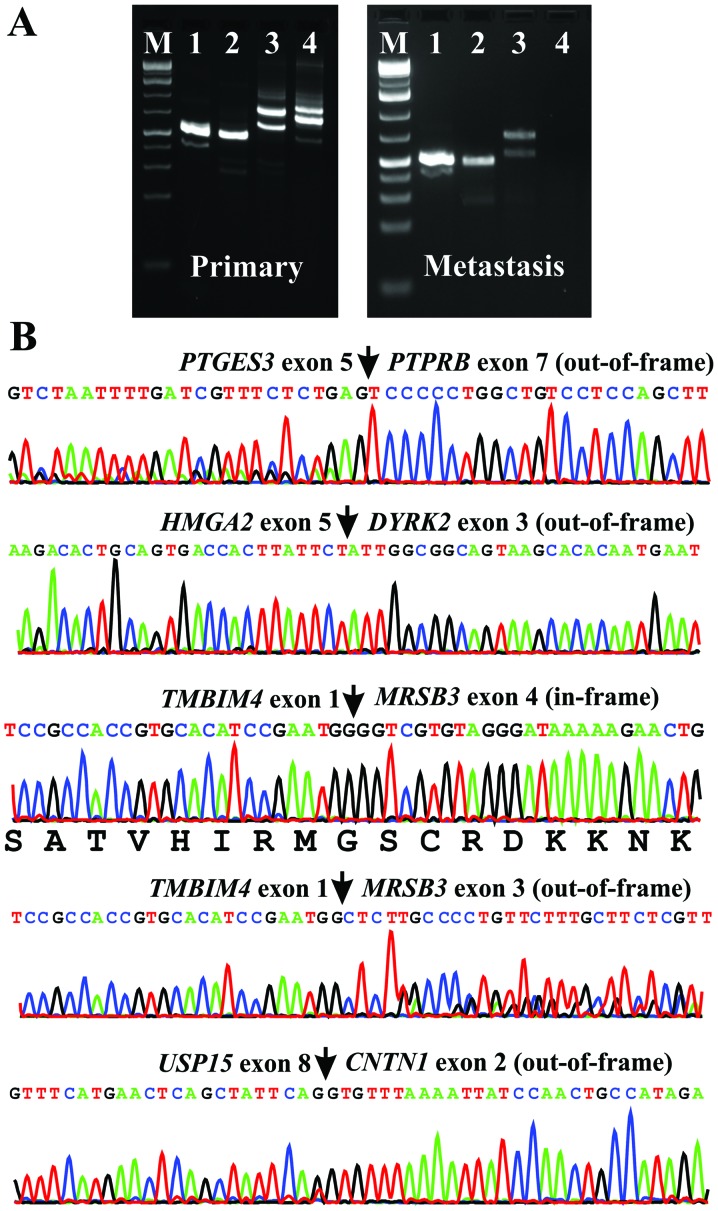
RT-PCR and Sanger sequencing of the PCR products. (A) Amplification of *PTGES3-PTPRB* (lane 1), *HMGA2-DYRK2* (lane 2), *TMBIM4-MSRB3* (lane 3) and *USP15-CNTN1* (lane 4) DNA fragments using cDNA from the primary tumor and metastasis. M, 1 Kb DNA ladder (GeneRuler, Fermentas). (B) Partial sequence chromatograms of the cDNA amplified fragment showing the junction position of the two partner genes in the *PTGES3-PTPRB*, *HMGA2-DYRK2*, two transcripts of *TMBIM4-MSRB3* and *USP15-CNTN1* fusion genes.

RT-PCR using cDNA from the primary tumor and subsequent direct Sanger sequencing verified the presence of *PTGES3-PTPRB*, *HMGA2-DYRK2*, *TMBIM4-MSRB3* and *USP15-CNTN1* chimeric transcripts (Fig. 3). PCR analysis of the metastasis amplified the fusion transcripts *PTGES3-PTPRB*, *HMGA2-DYRK2* and *TMBIM4-MSRB3* but not *USP15-CNTN1* (Fig. 3A). Sanger sequencing of the amplified products from the metastasis showed that they were identical to those amplified from the primary tumor.

**Figure 4:**
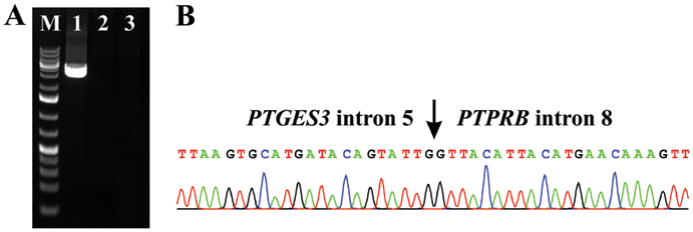
Extra-long-PCR for the genomic amplification of *PTGES3-PTPRB*, *HMGA2-DYRK2* and *TMBIM4-MSRB3* fusion DNA fragments. (A) Amplification of a genomic DNA fragment using the primers PTGES3-Int4-F1New and PTPRB-1602R DNA (lane 1). No *HMGA2-DYRK2* (lane 2) and *TMBIM4-MSRB3* (lane 3) genomic DNA fragments could be amplified. M, 1 Kb DNA ladder (GeneRuler, Fermentas). (B) Partial sequence chromatograms of the amplified DNA which showed that this fragment was a chimeric *PTGES3-PTPRB* genomic DNA. The breakpoints were located in intron 5 of *PTGES3* and intron 8 of *PTPRB*.

XL PCR using the primers PTGES3-Int4-F1New and PTPRB-1602R amplified a genomic DNA fragment (Fig. 4A) that was a chimeric *PTGES3-PTPRB* genomic DNA with the breakpoints located in intron 5 of *PTGES3* and intron 8 of *PTPRB* (Fig. 4B). XL PCR for amplification of genomic breakpoints of *HMGA2-DYRK2* and *TMBIM4-MSRB3* with the primers given above did not amplify any products (Fig. 4A). XL PCR with additional primer sets failed to amplify genomic hybrid *HMGA2-DYRK2* and *TMBIM4-MSRB3* fragments (data not shown).

**Figure 5:**
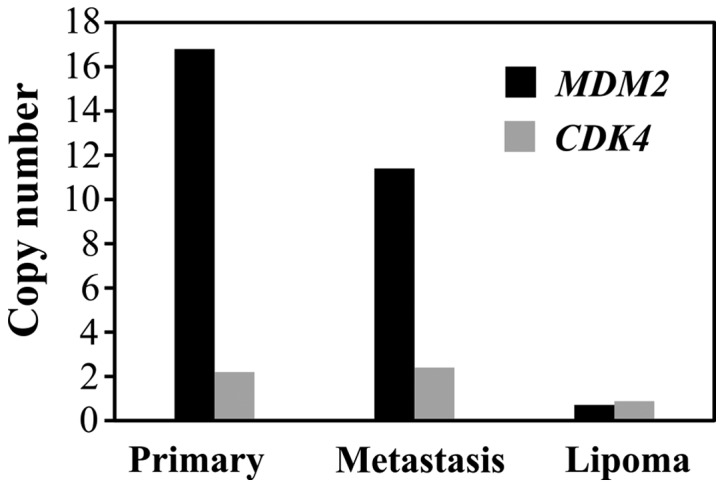
*MDM2* and *CDK4* amplification in the primary tumor, the metastasis and the control lipoma. In the primary tumor, the copy number of *MDM2* was 17.2 while the copy number of *CDK4* was 2.2. In the metastasis the copy number was 11.4 and 2.4 for *MDM2* and *CDK4*, respectively. *MDM2* and *CDK4* were not amplified in lipomas. The mean level for lipomas was 0.8 and 0.9 for *MDM2* and *CDK4*, respectively.

In the primary tumor, the copy number of *MDM2* was 17.2 whereas the copy number of *CDK4* was 2.2 (Fig. 5). In the metastasis, the copy number was 11.4 and 2.4 for *MDM2* and *CDK4*, respectively. The gene copy number (mean levels) of lipoma was 0.8 and 0.9 for *MDM2* and *CDK4*, respectively.

## Discussion

The spindle cell sarcoma we describe had a ring chromosome composed of material from chromosome 12. The molecular studies of primary tumor and metastatic cells showed both amplification of MDM2 and several fusion genes (four - *PTGES3-PTPRB*, *HMGA2-DYRK2*, *TMBIM4-MSRB3* and *USP15-CNTN1* in the primary tumor, whereas the first three, but not *USP15-CNTN1* were present also in the metastasis), all of them located on 12q (Fig. 2). This suggests that *USP15-CNTN1* was not expressed or was absent in the metastasis. *MDM2* amplification and the fusion transcripts *PTGES3-PTPRB*, *HMGA2-DYRK2*, *TMBIM4-MSRB3* were found both in the primary tumor and the metastasis examined, showing that the same clone set up these two tumor lesions. Which molecular change is the primary, or most primary, cannot be deduced from the data, but a role in initiation and/or progression is possible and indeed likely. The multiple copies of *MDM2* are not only correlated with the occurrence of ring chromosomes (16) but are located on the rings (17,18). It is reasonable to assume that in the present tumor both *MDM2* amplification and the fusion genes *PTGES3-PTPRB*, *HMGA2-DYRK2*, *TMBIM4-MSRB3* and *USP15-CNTN1* are located on the ring chromosome (Fig. 2) although lack of suitable material prevented us from determining this with certainty. The absence of *USP15-CNTN1* in the metastasis could reflect the instability of ring chromosomes. In the course of tumor progression, the ring(s) may break and re-join to form new rings. Thus, the rings in daughter cells can be different from each other and from the ring of the mother cell (19).

With exception of *HMGA2* and its multiple fusion genes in neoplasia (http://cgap.nci.nih.gov/Chromosomes/MSearchForm), none of the other three fusion genes we detected have been reported before in cancer (http://cgap.nci.nih.gov/Chromosomes/MSearchForm). The fusion transcripts are all out-of frame except one of the two alternative splicing transcripts of *TMBIM4-MRSB3* (Fig. 3). This suggests that the fusions play a role in the expression and/or regulation of genes by swapping promoter or 3′-untranslated region (UTR) and not by the formation of fusion proteins.

In *HMGA2-DYRK2* the fusion of *HMGA2* with *DYRK2* occurs at exon 5, 80 bp after the stop codon (position 1221 in sequence with accession number NM_003483.4) and exon 3 of *DYRK2* (position 601 in sequence with accession number NM_006482.2 or the position 452 of sequence with accession number NM_003583). Thus, only one of the eight *Let-a* target sites of the *HMGA2* 3′-UTR is present in the *HMGA2-DYRK2* fusion transcript (the one in position 1169 in sequence with accession number NM_003483.4) (20). DYRK2 belongs to a family of protein kinases whose members are presumed to be involved in cellular growth and/or development. The family is defined by structural similarity of their kinase domains and their capacity to autophosphorylate on tyrosine residues. DYRK2 has demonstrated tyrosine autophosphorylation and catalyzed phosphorylation of histones H3 and H2B *in vitro* (http://www.ncbi.nlm.nih.gov/nuccore/Nm_006482). In the *HMGA2-DYRK2* fusion transcript, the part of *DYRK2* coding the entire isoform 1 of DYRK2 protein (accession number NP_003574.1) is also present. It is therefore possible that the entire protein-encoding moiety of *DYRK2* is expressed either alone or as part of a bicistronic transcript encoding both HM GA2 and DYRK2 proteins under the influence of the *HMGA2* promoter. Bicistronic transcripts encoding two independent proteins have been reported in humans (21,22), and a bicistronic *CCND1-TROP2* mRNA chimera was even shown to have an oncogenic role in human cancer (23). The chimeric *CCND1-TROP2* mRNA, which independently translates both the cyclin D1 and TROP2 proteins, was isolated from human ovarian and mammary cancer cells and was found to be expressed in gastrointestinal, ovarian and endometrial tumors (23).

In *PTGES3-PTPRB*, the fusion of *PTGES3* with *PTPRB* occurs on exon 5 (position 670 in the sequence with accession number NM_006601.5) and exon 7 of *PTPRB* (position 1427 in the sequence with accession number NM_002837.4). PTGES3 functions as a chaperone which is required for proper function of the glucocorticoid and other steroid receptors (http://en.wikipedia.org/wiki/PTGES3). PTPRB is a member of the protein tyrosine phosphatase (PTP) family. PTPs are signaling molecules that regulate a variety of cellular processes including cell growth, differentiation, mitotic cycle and oncogenic transformation. This PTP belongs to the receptor type PTP and contains an extracellular domain, a single transmembrane segment, and an intracytoplasmic catalytic domain (http://en.wikipedia.org/wiki/PTPRB). The chimeric *PTGES3-PTPRB* transcript contains the part of *PTGES3* which codes for the putative Hsp90 binding site (conserved domain cd00237) and the part of PTPRB which catalyzes the dephosphorylation of phosphotyrosine peptides (conserved domain cd00047). The translation of a hypothetical bicistronic *PTGES3-PTPRB* fusion transcript would result in the production of two independent PTGES3 and PTPRB proteins with the above-mentioned functional domains.

The *MRSB3* gene codes for a protein which catalyzes the reduction of methionine sulphoxide to methionine (http://www.ncbi.nlm.nih.gov/gene/253827). This enzyme acts as a monomer and requires zinc as a cofactor. Four transcript variants have been identified encoding two different isoforms of the MRSB3 protein. Transcript variant 1 (accession number NM_198080) encodes the longer isoform 1 of MRSB3 and is found in the endoplasmic reticulum (ER), whereas transcript variants 2, 3 and 4 encode the same isoform 2 variant of MRSB3 which has shorter and distinct N-terminus compared to isoform 1 and is found primarily in mitochondria (24).

The result of *TMBIM4-MRSB3* fusion is that the expression of *MRSB3* comes under the control of the *TMBIM4* promoter. In the out-of-frame *TMBIM4-MRSB3* fusion transcript in which exon 1 of *TMBIM4* (nt 173 in the sequence with accession number NM_016056.2) is fused to exon 3 of *MSRB3* (nt 388 in the sequence with accession number NM_001193460.1), the result would be the production of the isoform 2 variant of MRSB3 which is localized to mitochondria. In the in-frame *TMBIM4-MRSB3* fusion transcript in which exon 1 of *TMBIM4* (nt 173 in the sequence with accession number NM_016056.2) is fused to exon 4 of *MSRB3* (nt 515 in the sequence with accession number NM_001193460.1), the result would be a chimeric TMBIM4-MRSB3 protein in which the first 32 amino acids of MRSB3 (MSPRRTLPRPLSLCLSLCLCLCLAAA LG SAQS) are replaced by the first 32 amino acids of TMBIM4 (MADPDPRYPRSSIEDDFNYGSSVASATVHIRM).

In a recent study, Nord *et al* (11) divided ring chromosomes in neoplastic cells into those with *MDM2* amplification and those without *MDM2* amplification. The amplification of *MDM2* is associated with co-amplification of a variety of potential driver oncogenes. Here we show that *MDM2* amplification may be associated with a variety of fusion genes. The sequence in which the various genetic events occur cannot be determined. The fact that both *MDM2* amplification and the fusion genes are found in both the primary tumor and metastasis indicate, however, that both play a role in tumorigenesis.

## Figures and Tables

**Table I:** Primers used for PCR amplification and sequencing.

Oligo name	Sequence (5′→3′)
PTGES3-416F1	CTC GG A GG A AGT GAT AAT TTT AAG C
PTPRB-1610R1	CTG TAG CCA TGT ATT TTC GTC CAG
HMGA2-982F1	CAA GAG TCC CTC TAA AGC AGC TCA
DYRK2-733R1	CCG TTT TGC CCA CTG TTG TAA G
DYRK2-709R1	GCC CAT TTG GTT GTG TCG TGA GCA C
DYRK2-651R	TTG AAC CTG GAT CTG TCC GTG AGC G
TMBIM4-25F1	AGG AGG CGG TTG CGG TTA GTG
MSRB3-501R	TTT CCA CCC TGT GCA TCC CAT AG
USP15-655F1	CCA GAC AGC ACC ATT CAG GAT GC
CNTN1-413R	CTG GCT CGT GCC CTA CAG TTG AGT
PTGES3-Int4-F1New	CCT TGG TCA GAA ACG GAG CTG GTC AA
PTGES3-Int4-F2New	AAT GCT TGC TGC ACT CCA GCC TGG
PTPRB-1602R	CCA TGT ATT TTC GTC CAG GCA CCA GG
PTPRB-1490R	TCC CAT TCT GCT ACA GG GTC TGC C
HMGA2-Int4-F1	CCT CTG CAC TGT TGG CAA GAG CAG C
HMGA2-Int4-F2	CGA TTG AGC GTC ATG GCT GTG CC
TMBIM4-55F1New	CGG TAG GGG TGC TGT TGC CAT CAT G
TMBIM4-121F1New	CGA CTT CAA CTA TGG CAG CAG CGT GG
MSRB3-350R1New	TGC AGT CTC ACT GTG CTT CCA CAG CTG A
MSRB3-261R1New	ACC ACG GTG GCC ATC TGG CTT ATG TC
